# Structural Design of a Nanogel Reaction Device with Emphasis on Temperature-Field Uniformity

**DOI:** 10.3390/ma19071298

**Published:** 2026-03-25

**Authors:** Zihao Tang, Mingzhe Wang, Jialong Liu, Zijia Zeng, Jing Guo, Xiaoming Yu, Lili Li

**Affiliations:** 1 School of Materials and Metallurgy, University of Science and Technology Liaoning, Anshan 114051, China; 17512810985@163.com (Z.T.); 13804229855@163.com (Z.Z.);; 2 School of Materials Science and Engineering, Shenyang Ligong University, Shenyang 110180, China; xmyu@sylu.edu.cn

**Keywords:** TRIZ, nanogels, temperature-field uniformity, PTC self-limiting heating, particle size distribution

## Abstract

Thermosensitive nanogels are highly sensitive to temperature fluctuations, making precise thermal control critical for uniform particle formation and consistent product quality. This study introduces a TRIZ-guided reactor design that integrates material-level self-feedback heating with structurally homogenized heat transfer, shifting temperature regulation from conventional multi-point feedback to intrinsic physical adaptation. Experimental evaluation demonstrated that the optimized system reduced the maximum temperature difference from 6.5 °C to 3.2 °C and decreased the standard deviation from 3.9 °C to 1.8 °C, resulting in improved reaction stability. Correspondingly, monomer conversion increased from 90% to 95%, and the particle size distribution narrowed, with PDI decreasing from 0.32 to 0.18. The energy consumption per unit mass of reactant also decreased from 3.6 kJ·g^−1^ to 2.5 kJ·g^−1^. These results indicate that the TRIZ-based self-feedback approach effectively enhances temperature uniformity, reaction control, and energy efficiency, providing a transferable strategy for temperature-sensitive polymerization and nanomaterial synthesis.

## 1. Introduction

Nanogels have been addressed as promising candidates for drug delivery, gene therapy, and imaging owing to their high-water-content networks, capacity to load diverse active molecules, and amenability to surface engineering [1,2,3]. Among them, thermosensitive nanogels can precisely tune the phase-transition temperature (e.g., the lower critical solution temperature, LCST) by adjusting polymer composition, thereby enabling temperature-triggered encapsulation and release [4,5,6]. Recent studies have further shown that the network architecture, crosslinking density, and surface functional groups of nanogels significantly influence their response kinetics and biological performance, thereby increasing the demand for controllability in the synthesis process [7,8,9].

To improve process controllability, substantial international efforts have shifted toward process intensification and reactor engineering. Microfluidic and continuous-flow approaches can deliver rapid mixing and enhanced heat transfer at small scales while enabling precise control of key parameters such as temperature and flow rate, and are therefore considered effective for producing more uniform nanostructured products [10]. However, when scaling up or retrofitting existing batch equipment, practical obstacles persist, including limited throughput in microfluidic systems, clogging risks, increased pressure drop at higher viscosities, and the complexity of thermal management [11]. Meanwhile, many production scenarios still rely on jacketed reactors or agitated batch and semi-batch systems, in which temperature-field nonuniformity and hotspot formation are even more pronounced. Related studies indicate that, in aggregation reactors, under strongly exothermic conditions and limited heat transfer, temperature and composition disturbances can be amplified and may exhibit unstable behavior, suggesting that reliance solely on the traditional “temperature measurement–control–heating” feedback loop is insufficient to suppress spatial temperature gradients at the source [12,13,14]. Current engineering improvements often involve adding measurement points, adopting more complex control strategies, and implementing zoned operation, but these measures typically increase system complexity, calibration and maintenance costs, and the number of potential failure points; moreover, when measurement points are not sufficiently representative, local temperatures may be misinterpreted as the bulk temperature, potentially leading to overshoot and localized overheating. In contrast, intrinsically embedding safety and temperature uniformity into the heat source or structural body aligns more closely with the engineering ideal, and positive temperature coefficient (PTC) heating elements are attractive in this regard because their electrical resistance increases with temperature, enabling automatic power reduction as the target temperature range is approached; accordingly, their self-limiting behavior and tolerance to voltage fluctuations can reduce overheating risk and simplify external control circuitry [15,16,17].

Based on the above background, this paper proposes an innovative solution to the “temperature uniformity–complexity” contradiction using the TRIZ theoretical framework. Through functional analysis and engineering contradiction resolution, a new reactor design combining PTC self-limiting heating elements with a thermal diffusion buffer structure is proposed. The PTC self-limiting heating element automatically reduces heat input as it approaches the target temperature zone through temperature-dependent power attenuation, thereby suppressing overheating and preventing reaction instability caused by temperature overshoot. Comparative experimental results verify the effectiveness of this design scheme in improving temperature field uniformity, enhancing reaction stability, and reducing energy consumption. The results show that the “self-feedback temperature-limited and structurally isothermal” design scheme significantly improves the quality and energy efficiency of nanogels without increasing system complexity. The innovation of this study lies in identifying the core contradictions in temperature control through the TRIZ framework and proposing a temperature control design based on physical-level adaptive regulation and structural optimization. This provides new insights and transferable design paradigms for the engineering of temperature-sensitive polymerization reactions.

## 2. Materials and Methods

### 2.1. Materials and Thermosensitive Performance

The nanogel reaction device targets the entire reaction system involved in nanogel particle formation. As listed in Table 1, the reaction medium consists of the monomer N-isopropylacrylamide, the crosslinker N,N′-methylenebisacrylamide, the initiator ammonium persulfate, the stabilizer sodium dodecyl sulfate, and deionized water as the solvent. The main reaction conditions in this experiment include reaction temperature, stirring rate, and reagent addition method. The temperature program follows a two-step control strategy. The temperature is first increased linearly from 25 °C to 40 °C to rapidly pass through the heating and activation stages. It is then adjusted to 35 °C and maintained for 2 h. During the reaction, magnetic stirring is applied at 300 rpm to ensure efficient mixing and heat transfer. The working volume is 80 mL, corresponding to 80% of the total reactor volume and providing sufficient reaction space. At the beginning of the reaction, the monomer NIPAM, the crosslinker MBAA, and the initiator APS are added together to ensure a stable and homogeneous initial reaction environment. The concentration of MBAA is 5 wt% relative to the mass of NIPAM. All reagents are prepared from high-purity raw materials to ensure reaction controllability and result reproducibility. The nanogel reaction device regulates the uniformity and stability of these fields through temperature control, stirring, and heat exchange. These factors influence the nucleation–growth–crosslinking pathway and determine the particle size distribution and network structure [18].

**Figure 1:**
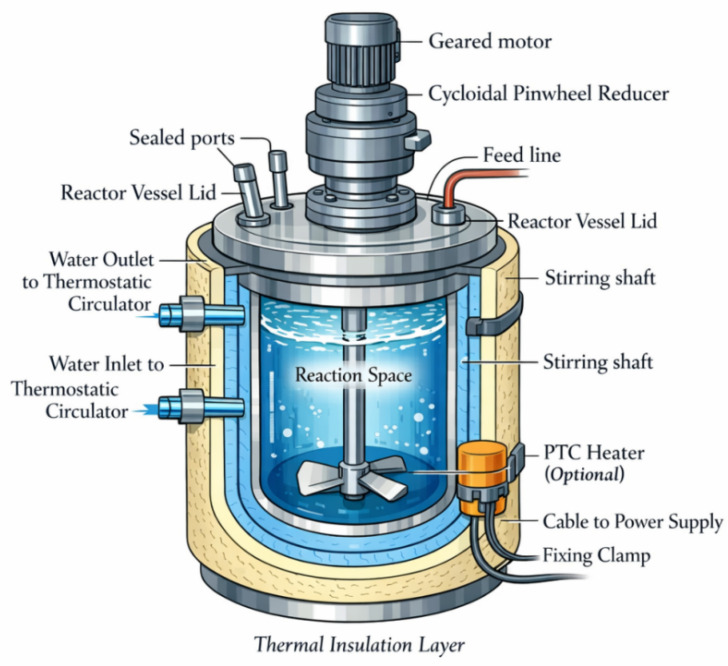
Schematic diagram of a conventional nanogel device.

Analysis of the conventional nanogel preparation apparatus (Figure 1) indicates that the key limitation is not temperature-control accuracy per se, but rather the ability to suppress temperature disturbances. The reaction system is highly sensitive to its temperature history near the phase-transition temperature range; even slight spatial or temporal fluctuations can be amplified through reaction kinetics and through coupling among viscosity, heat transfer, and mixing. These effects ultimately manifest as deviations in the nucleation and growth pathways, nonuniform crosslinking structures, and a broadened particle size distribution. In other words, the critical performance lies in the device’s ability, under a prescribed target temperature trajectory, to minimize the sensitivity of the system’s effective reaction rate (or equivalent conversion pathway) to temperature disturbances. To this end, a temperature-sensitivity amplification factor is introduced, and a device temperature-sensitivity performance index is constructed as follows.

Temperature measurements were performed using eight K-type thermocouples installed inside the reactor. Duplicate thermocouples were placed at four locations to evaluate measurement consistency. Before the experiments, all thermocouples were calibrated. Systematic errors were corrected using standard temperature references, including an ice–water mixture at 0 °C and boiling water at 100 °C. After calibration, the measurement uncertainty of each sensor was approximately ±0.5 °C. According to ASTM E230 [19], the standard limits of error for K-type thermocouples over the range from room temperature to 400 °C are typically ±2.2 °C for standard-grade sensors and ±1.1 °C for premium-grade sensors. The overall measurement uncertainty was determined from the calibration records and the accuracy of the data acquisition system. This uncertainty was taken into account in the subsequent calculations of $∆T_{max}$ and $\sigma_{T}$.

Statistical analysis was performed on the temperature data collected from multiple measurement points inside the reactor. The instantaneous temperature deviation (RMS) was defined as follows:

(1) $$\sigma_{T}(t)=\sqrt{\frac{1}{N}\sum_{i=1}^{n}{\left(T_{i}\left(t\right)-\bar{T_{i}}(t)\right)}^{2}}$$

Here, $T_{i}$ is the temperature at the ith measurement point at time t, $\bar{T_{i}}$ is the average temperature of all measurement points, and N is the total number of measurement points. This definition reflects the temperature uniformity and transient fluctuations inside the reactor. A represents the maximum temperature difference in the reactor temperature field during the experiment. It is defined as the extreme temperature difference among different measurement points at the same time. This parameter was used to quantify temperature-field uniformity. The specific definition is as follows:

(2) $$∆T_{max}=max(T_{1}(t),T_{2}(t)\ldots T_{i}(t))-min(T_{1}(t),T_{2}(t)\ldots T_{i}(t))$$

To assess the measurement uncertainties of $∆T_{max}$ and $\sigma_{T}$, the standard uncertainty propagation method recommended in the Guide to the Expression of Uncertainty in Measurement (GUM) was adopted. The uncertainty of each thermocouple included the intrinsic sensor accuracy, calibration error, and data acquisition system error. The uncertainty of $∆T_{max}$ was obtained by combining the temperature uncertainties of the maximum and minimum measurement points. The uncertainty of $\sigma_{T}$ was calculated using the error propagation formula:

(3) $$u_{\sigma_{T}}=\frac{1}{\sigma_{T}}\sqrt{\frac{1}{N^{2}}\sum_{i=1}^{N}(T_{i}\left(t\right)-\bar{T_{i}}(t))u_{i}^{2}}$$

The reaction rate changes with temperature according to the standard Arrhenius [20] form:

(4) $$k(T)=Aexp(-\frac{E_{\alpha }}{RT})$$

where A is the pre-exponential factor, $E_{\alpha }$ is the activation energy, R is the gas constant, and T is the absolute temperature. After linearization, it can be expressed as:

(5) $$lnk(T)=lnA-\frac{E_{\alpha }}{R}\frac{1}{T}$$

This form, combined with experimental temperature control data, can be used to quantitatively analyze the effect of temperature on the reaction rate. Based on temperature deviation $\sigma_{T}$, an instantaneous temperature control performance function is defined as:

(6) $$\Gamma (t)=\frac{\sigma_{T}(t)}{T_{set}}$$

where $T_{set}$ is the set temperature of the reactor, which quantifies the uniformity and stability of the temperature at any given moment. Finally, by integrating the instantaneous performance function within the critical reaction time window [$t_{0}$, $t_{f}$], the temperature sensitivity performance index is obtained:

(7) $$\eta TS=\frac{1}{t_{f}-t_{0}}\int_{t_{0}}^{t_{f}}\Gamma (t)dt$$

$\sigma_{T}$ reflects the overall dispersion of temperatures measured at different points relative to the average temperature. $∆T_{max}$ represents the extreme difference between the highest and lowest temperatures at a given moment. ηTS is a comprehensive indicator obtained by averaging the normalized temperature fluctuation level over the entire process with respect to the set temperature, $T_{set}$. $\sigma_{T}$ and $∆T_{max}$ serve as direct indicators of temperature-field uniformity. By contrast, ηTS evaluates temperature control deviation over time on the basis of $\sigma_{T}$. This parameter can be directly calculated from experimental temperature data and used to compare reactor performance under different temperature control strategies, such as conventional heating and PTC self-limiting heating. It quantitatively reflects improvements in temperature overshoot, stabilization time, and spatial temperature uniformity. It also provides guidance for reactor design and optimization.

### 2.2. Nanoparticle Testing

**Figure 2:**
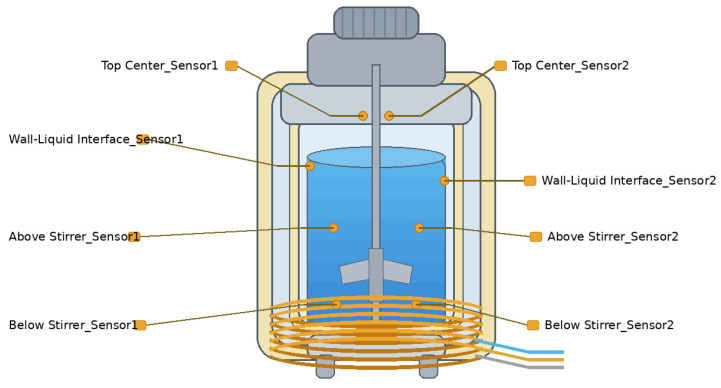
Distribution of K-type thermocouples.

In this study, eight K-type thermocouples were used for multi-point temperature monitoring to ensure the uniformity of the temperature field during the nanogel synthesis reaction. The placement of the thermocouples was optimized based on the reactor’s structure. The specific arrangement is shown in Figure 2. Two thermocouples are installed at the axial center of the reactor, positioned at the upper and lower parts, respectively, to monitor temperature changes in the vertical direction. Two thermocouples are placed between the liquid and the reactor wall to monitor the temperature difference between them. Another two thermocouples are positioned above and below the stirrer to monitor potential local temperature fluctuations during stirring. The remaining two thermocouples are installed at the middle and bottom of the outer wall to monitor temperature and heat loss. Temperature data are collected in real-time using the NI USB-9211A data acquisition module (±0.1 °C), with a sampling frequency of 1 Hz, ensuring accurate recording of temperature changes every second. The data acquisition module is connected to the computer and utilizes self-programmed data processing software for real-time monitoring. The temperature data at each time point are used to verify the uniformity of the temperature field inside the reactor. This ensures that temperature fluctuations remain within the preset target range and prevents the impact of excessively high or low temperatures on nanogel synthesis, especially during the critical temperature windows for nucleation, crosslinking, and polymerization reactions.

During the nanogel synthesis reaction, multiple sampling time points were designed to avoid transient temperature interference with the particle structure. At each sampling point, a small amount of sample was immediately taken from the reaction system and quickly cooled to room temperature or with a cooling medium to “quench” the reaction, thereby terminating the process. To ensure that the transparency and scattering intensity of the dispersed system met the requirements for dynamic light scattering tests, all samples were serially diluted using deionized water or a buffer with equivalent ionic strength. To reduce the interference of dust and micro-aggregation on the test results, all samples were briefly dispersed using ultrasound, and large particle impurities were removed with microporous filters, ensuring the uniformity and stability of the samples. The particle size and particle size distribution were characterized using the Malvern Zetasizer Nano ZS Dynamic Light Scattering (DLS, ±2%, Malvern Panalytical Ltd., Malvern, UK), with a particle size range of 1–1000 nm. The DLS test used a backscattering detection mode with a scattering angle of 173°, a test temperature of 25 °C, deionized water as the dispersion medium, and the instrument’s built-in water model for viscosity. Each sample was measured at least three consecutive times. The Z-average particle size, PDI, representative intensity distribution curve, and autocorrelation function were recorded to evaluate the consistency and dispersion stability of the reaction products. To further investigate the relationship between particle formation mass and reaction conversion, some samples were subjected to freeze-drying or vacuum drying. The changes in the characteristic functional group absorption peaks were then measured using an FT-IR spectrometer (Thermo Scientific Nicolet iS5, Waltham, MA, USA). The FTIR test range was 4000–500 cm^−1^, with a resolution of 4 cm^−1^, and 32 scans were accumulated. This paper characterizes the reaction conversion based on the relative changes in the monomer double bond-related bands before and after polymerization, as well as the amide bands. The C=C-related absorption band at about 1620–1640 cm^−1^ was used as the diagnostic band, and the amide I band (around 1650 cm^−1^) or amide II band (around 1540 cm^−1^) served as the internal reference band. After baseline correction of the spectra, the peak area ratio was calculated using the peak area integration method, and the conversion rate was estimated accordingly. To ensure comparability, all samples underwent the same baseline subtraction range, integration interval, and spectral preprocessing procedure. All samples were from independently synthesized batches, and the experimental results are expressed as mean ± standard deviation.

### 2.3. TRIZ Method

**Figure 3:**
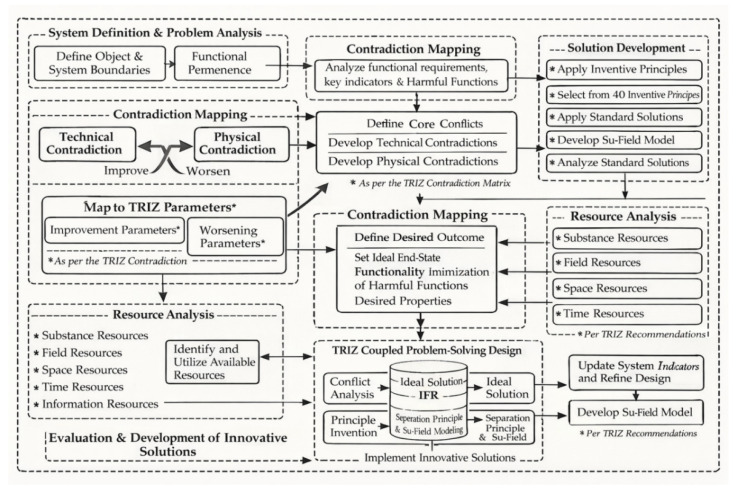
TRIZ problem-solving approach.

Within the TRIZ framework, the solution pathway can be summarized as “system definition—problem essence identification—contradiction refinement—idealization and resource-driven analysis—model-based solution development—principle implementation,” as shown in Figure 3. First, the research object was defined as the nanogel synthesis reaction apparatus by delineating system boundaries and subsystem components and identifying beneficial functions—stirring to promote mixing and heat transfer, heating to reach the target temperature zone, and feed sealing to ensure process execution—as well as key harmful functions, including the limited representativeness of temperature measurement points that can cause control deviations, localized overheating and temperature gradients, and increased complexity and maintenance burden arising from additional closed-loop links. This functional analysis pinpointed the core conflict between the need for temperature uniformity and increased system complexity and side effects. The problem was then translated into technical and physical contradictions, forming an “improvement–deterioration” pair that can be mapped to TRIZ parameters, with the ideal final result (IFR) defined as achieving temperature-zone stability and uniformity with minimal additional cost and side effects. Next, a resource analysis was conducted by inventorying available in-system and external resources across the dimensions of material, field, space, time, and information, thereby guiding solution generation toward the use of local resources. Furthermore, using the Su-Field model, the problem was abstracted into an action system in which a thermal field acts on a material. For typical scenarios such as insufficient effect, harmful effect, or overreliance on measurement-point information, solutions were generated by applying the 76 standard solutions and relevant inventive principles. Finally, the selected principles were translated into feasible structural modification concepts, thereby closing the loop from methodological reasoning to implementable engineering solutions.

## 3. Results and Discussion

### 3.1. TRIZ Solution

#### 3.1.1. Function Analysis

To support subsequent TRIZ contradiction modeling and resolution, the research object was defined as a nanogel synthesis reaction device, with the system boundary encompassing core elements such as the vessel, stirring mechanism, heat input, heat-loss suppression, and sealed sampling. The power supply and production environment (heat dissipation conditions, operating fluctuations, and operator-related factors), together with feedstocks and process parameters, were treated as supersystem inputs and constraints. Key internal subsystems include the mixing subsystem, the feeding and sealing subsystem, and the thermal-control subsystem.

Under the above system structure, a functional analysis of the nanogel reaction device was conducted. The core beneficial functions include the following: the reaction medium and promotes convection to enhance mixing uniformity and convective heat transfer; heat to the vessel to reach and maintain the target reaction temperature zone; and the safe addition of liquid reagents while maintaining system sealing. The key harmful effects are primarily associated with the limited representativeness of temperature-sensor measurement points with respect to the system’s actual temperature field. Variations in sensor placement can cause measured values to deviate from the bulk temperature distribution, resulting in temperature-control errors and potentially inducing localized overheating or temperature gradients, which can in turn perturb the polymerization process and compromise the quality and stability of thermosensitive nanogel systems. Meanwhile, the closed-loop control chain (measurement–signal–control–actuation) comprises multiple links, introducing complexity and maintenance burdens associated with sensor placement, calibration, and dynamic response delays, which constitute typical side-effect functions. To facilitate subsequent conflict extraction, the main functional interactions are summarized in Table 2.

#### 3.1.2. Contradiction Analysis

Functional analysis indicates that the primary source of temperature-control deviations is the limited representativeness of measurement-point temperatures with respect to the spatial temperature field, which can lead to temperature gradients. In parallel, expanding the external closed-loop control chain (measurement–signal–control–actuation) can introduce complex side effects, including increased layout complexity, greater calibration demands, response delays, and higher maintenance burdens. Together, these factors create a typical engineering contradiction in which performance improvement coexists with increased system complexity and associated side effects. A summary of the engineering and physical contradiction analysis for nanogel synthesis reaction equipment is provided in Table 3. As shown in the table, the engineering conflict centers on improving temperature-field uniformity and process stability; however, conventional approaches often pursue these goals by adding sensing, control, and actuation steps, which increases system complexity and maintenance burden and may elevate the risk of overheating. Furthermore, the physical contradictions reveal conflicting “both…and…” requirements regarding heat-input intensity and the representativeness of temperature measurements [21,22].

#### 3.1.3. IFR and Resource Analysis

**Figure 4:**
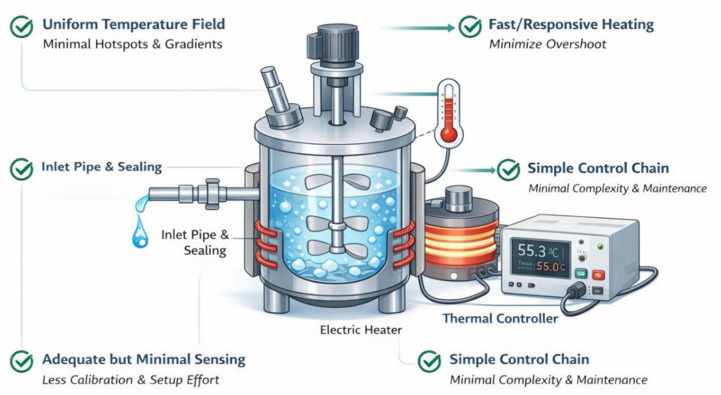
Ideal diagram of the nanogel synthesis device.

Analysis of the engineering and physical contradictions indicates that nanogel synthesis requires higher temperature-field uniformity and more stable maintenance of the target temperature zone to mitigate localized overheating, temperature gradients, and the resulting process fluctuations. However, conventional improvement pathways often rely on adding measurement points and strengthening external closed-loop control and actuation, which increases device complexity, raises setup and calibration burdens, reduces reliability and maintainability, and further amplifies potential sources of control deviation. Under these contradictory constraints, the ideal outcome should unify performance improvement with cost minimization, that is, achieve the target function without introducing significant new side effects [23,24]. Accordingly, the IFR derived from the TRIZ-based analysis is shown in Figure 4.

TRIZ resource analysis emphasizes prioritizing the use of existing resources within the system, between systems, and in the supersystem to achieve the IFR with minimal additional cost [25,26]. Based on the device idealization shown in Figure 3, available resources can be categorized into five types—material, field, space, time, and information—as summarized in Table 4.

#### 3.1.4. Su-Field Modeling and Standard Solution 76

**Figure 5:**
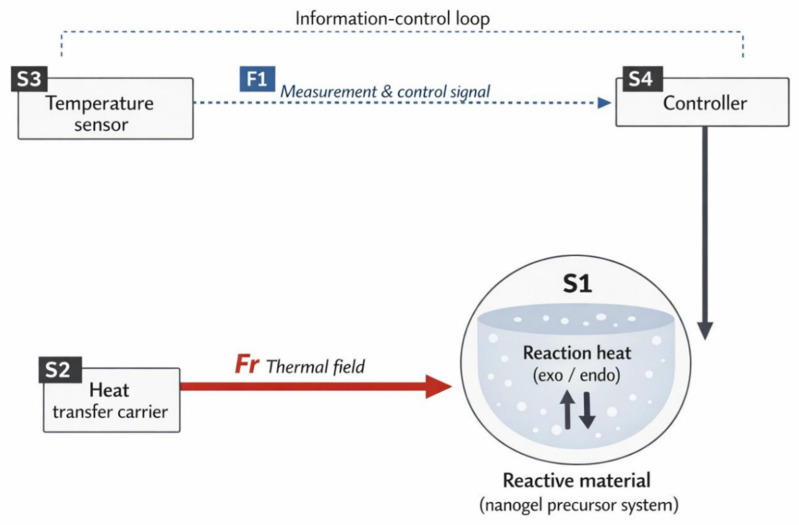
Su-Field model.

Based on the functional analysis, the temperature-control issue can be abstracted as a typical Su-Field system in which a thermal field acts on the reactant material, as shown in Figure 5. The system comprises substance S_1_, the reactant material (the nanogel precursor system containing monomers, solvent, initiator, etc.); substance S_2_, the heat-transfer carrier/acting body (e.g., the reactor wall, heating element, and jacket medium, depending on the existing apparatus configuration); and field F, the thermal field F_r_ jointly formed by external heat input and the reaction’s exothermic/endothermic processes. In conventional externally closed-loop temperature-control scenarios, additional measurement and control elements are involved, including substance S_3_, temperature sensors and their measurement points (providing temperature information); information field F_1_, measurement information and control signals (acquired by the sensor and transmitted to the controller); and substance S_4_, controllers and actuators (e.g., temperature controllers and electric-heater power regulation units).

The existing setup can be regarded as a coupled system comprising a thermal-field subsystem and an information closed-loop subsystem. The key issue is that the temperature information provided by S_3_ is not sufficiently representative, which can cause S_4_ regulation to deviate from the actual temperature field, resulting in localized overheating or temperature gradients. Meanwhile, the information closed-loop subsystem increases device complexity and maintenance burden. Therefore, the objective of the standard solution can be stated as transforming the action of F_r_ on S_1_ from locally nonuniform, overly strong, or insufficient to globally controllable and uniform—without significantly increasing system complexity—while reducing reliance on the representativeness of S_3_ measurement points.

In this regard, the 76 standard solutions are commonly organized into groups such as “improving/building the Su-Field system,” “eliminating harmful effects,” “enhancing insufficient effects,” “introducing measurement and feedback,” and “increasing controllability and ideality.” Based on the problem types described above, these solutions can be mapped onto four highly relevant standard-solution directions (corresponding to multiple groups of standard-solution entries).

**Route A:** Introduce or modify S_2_, and/or introduce an intermediate substance S_x_, to make the thermal action more uniform (i.e., enhance an insufficient effect).

**Route B:** Separate, divert, or eliminate harmful effects (i.e., eliminate harmful effects).

**Route C:** Introduce a self-regulating/self-feedback heat source so that the information closed-loop is shifted toward physical-level self-adaptation, thereby reducing dependence on S_3_ (measurement-point representativeness).

**Route D:** Construct a “measure–correct” substance/field compensation pathway to make information more reliable and/or reduce sensitivity to single-source information.

Without prescribing a specific structure a priori, Routes A–D can be further developed into comparable conceptual schemes as follows:

**Scheme 1:** Utilize the system’s material resources (e.g., vessel wall, jacket medium, insulation structure) to build more uniform heat-transfer and thermal-buffer pathways, thereby reducing temperature gradients.

**Scheme 2:** Utilize the system’s field resources (electrical power input) together with the temperature-dependent properties of the selected materials/structures, such that heat input is automatically adjusted with the temperature state, enabling physical self-regulation with conditional separation (i.e., high input at low temperature and low input at high temperature).

**Scheme 3:** Reduce dependence on the control chain and on measurement-point representativeness by shifting the approach from “control-correction” based on temperature measurement points to a more physically adaptive mechanism (i.e., addressing the representativeness issue of single-point temperature information), or by introducing an additional low-cost information channel (e.g., heating power/current sensing) to assist in system-state estimation.

#### 3.1.5. Mapping of the Invention Principle

Based on the preceding functional analysis and Su-Field modeling (i.e., a system-level description of thermal fields acting on reactive materials), TRIZ methods can map the extracted engineering and physical contradictions to the corresponding inventive principles, thereby yielding feasible structural improvement solutions for practical device design. Building on the engineering and physical contradiction analysis in Section 2.2, this section systematically applies the TRIZ 76 standard solutions across different design stages to address the challenge of improving temperature uniformity while reducing control complexity, as summarized in Table 5.

Based on the inventive principles identified in Table 5, the following design proposals are proposed as practical engineering improvement pathways:

**Pathway 1:** Design multiple independent heating zones within the reactor, each equipped with a local temperature sensor, to optimize spatial heat-flux distribution. The heat input of each zone is adjusted according to its specific thermal demand, thereby mitigating measurement-point bias and preventing overheating typical of conventional single-point temperature control.

**Pathway 2:** In addition to conventional single-point temperature sensing, deploy multiple temperature measurement points at different reactor locations and implement advanced PID-based control to improve temperature-field uniformity and reduce the risk of localized overheating.

**Pathway 3:** Apply high-thermal-inertia materials or thermal-buffer structures on the reactor wall to passively slow temperature rise during heating, thereby reducing overshoot and decreasing reliance on external closed-loop correction.

**Pathway 4:** Use PTC materials to fabricate the reactor heating element so that heating power is automatically adjusted; once the preset temperature is approached, heat input decreases inherently, thereby avoiding temperature overshoot and localized overheating.

**Pathway 5:** Introduce an interlayer between the reactor wall and the external environment and fill it with an inert gas or a heat-transfer fluid (e.g., heat-transfer oil). This configuration buffers heat transfer, optimizes heat-flux distribution, and enhances temperature-zone stability.

### 3.2. Optimal Solution Selection and Working Principle

Based on the TRIZ analysis, the pathway most consistent with the IFR and the guidance of the 76 standard solutions is to shift the control function from the external information loop (S3–F1–S4) to physical-layer self-feedback, such that the action of the thermal field on the reactive material becomes inherently self-limited and stabilizes as the system approaches the target temperature zone. Accordingly, the optimal solution primarily follows Pathway 4. At the same time, meeting the requirements for spatial thermal-field uniformity and energy efficiency necessitates leveraging existing in-system resources for heat diffusion, thermal buffering, and heat-loss suppression. Therefore, supplementing the design with Pathway 5 can further reduce temperature gradients and environmental disturbances, yielding an optimally integrated solution featuring self-feedback temperature control and a structurally uniform temperature field. In contrast, Pathways 1 and 2 essentially rely on zonal control and multi-point temperature measurement with feedback regulation to improve temperature uniformity. Although this can enhance measurement and control accuracy, it directly increases system complexity and maintenance costs and does not fundamentally eliminate the risk of control deviation arising from measurement points that are not representative of the overall temperature field. Pathway 3 primarily emphasizes thermal-capacitance buffering, which can attenuate fluctuations but is difficult to realize as an adaptive temperature-limiting strategy across varying operating conditions and to reduce dependence on measurement points; therefore, its ideality is limited in the TRIZ sense.

**Figure 6:**
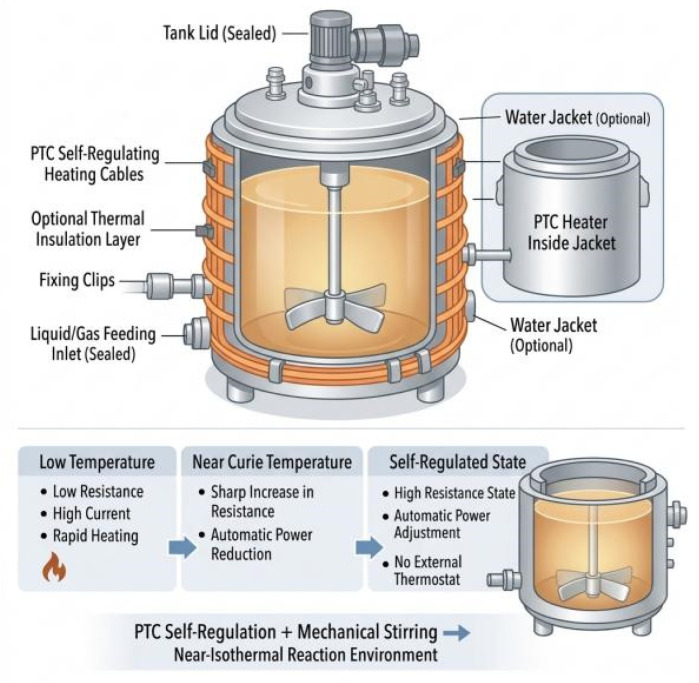
Self-regulating nanogel reaction system.

Ultimately, the approach adopted in this paper falls within a “material-level, self-feedback thermal input with structurally homogenized heat transfer” scheme. A PTC self-regulating heating element is used to achieve power self-adaptation without an external thermostat, and insulation layers, water interlayers, and interface coatings are incorporated to enhance heat-transfer stability and temperature-field uniformity. This design reduces reliance on measurement points and mitigates the risk of localized overheating, as shown in Figure 6.

### 3.3. Key Structural Parameters and Manufacturability

**Figure 7:**
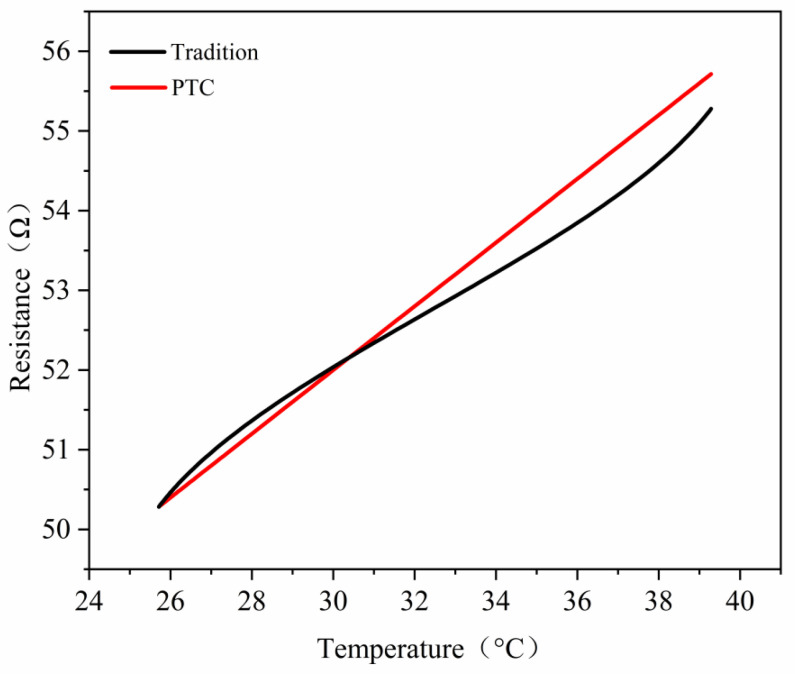
Resistance-Temperature R(T) curve of conventional heating system and PTC self-limiting heating system.

**Figure 8:**
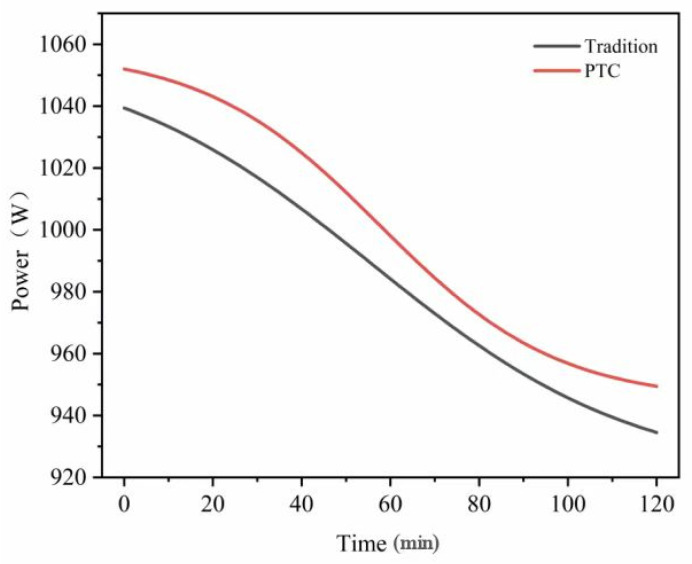
Power-Time P(t) curves of conventional heating system and PTC self-limiting heating system.

To clarify the role of the PTC self-limiting characteristic at actual reaction temperatures, this study measured the resistance–temperature, R(T), curves of the BARTEC HTSB PTC self-limiting heating cable over the range of 25–50 °C, as shown in Figure 7. The power–time, P(t), curves were also measured over the same temperature range, as shown in Figure 8. The cable had an outer diameter of 12.1 mm, a rated power of 30 W/m, and was powered by a 230 V AC supply. The R(T) curve was obtained by placing the cable in a constant-temperature chamber and increasing the temperature from 25 °C to 50 °C at a rate of 1 °C/min. The instruments used were a Julabo F25 constant-temperature chamber (JULABO GmbH, Seelbach, Germany) and a Keysight 34461A digital multimeter (Keysight Technologies, Santa Rosa, CA, USA). The P(t) curve was recorded using a power analyzer (Yokogawa WT310E, Yokogawa Electric Corporation, Tokyo, Japan), which calculated the power from the time-dependent current variation at a sampling frequency of 1 Hz. The cable was fixed to the outer wall of the reactor with soft iron band clamps. The clamp spacing was maintained at 150–250 mm to ensure good contact.

During the heating-up phase (25–40 °C), conventional heating systems exhibit a slow temperature response. The temperature curve is relatively gradual. In the middle stage of heating, the temperature remains below the setpoint, which results in sustained high power input. The spatial temperature distribution is therefore prone to delay and local deviations. The corresponding R(T) curve shows that the resistance increases approximately linearly with temperature. The variation remains limited in the range of 35–40 °C. The resulting power attenuation is small. This behavior cannot effectively suppress temperature overshoot. The P(t) curve further indicates significant power fluctuations throughout the heating stage. The heating rate is constrained by thermal inertia. This condition may lead to local overheating in the reaction system or uneven reaction rates.

In contrast, the PTC self-limiting heating system shows clear advantages over the same temperature range. The R(T) curve exhibits a smooth S-shaped profile. The resistance changes gradually at lower temperatures and increases rapidly at higher temperatures. This characteristic enables fine power adjustment in the 35–40 °C range. The heating rate remains moderate while temperature overshoot is suppressed. The P(t) curve shows that the PTC system provides sufficient power during the initial heating stage. The temperature therefore approaches the target value rapidly. The power gradually decreases as the temperature approaches the setpoint. This response produces self-limiting regulation. The temperature field becomes more stable and uniform.

The suppression of temperature overshoot originates from the positive temperature coefficient behavior of the PTC material. As the temperature approaches the upper setpoint, such as 40 °C, the cable resistance increases nonlinearly. The output power then decreases gradually (see Figure 7 and Figure 8). This automatic adjustment offsets the continued temperature rise caused by thermal inertia. As a result, the heating process becomes smoother. The PTC self-limiting mechanism stabilizes and homogenizes the temperature field by fine-tuning the power input in key temperature zones. This behavior prevents local overheating and transient overshoot. This feature is particularly important for thermosensitive polymer systems such as nanogels. It ensures uniform temperature-controlled conditions during nucleation, polymerization, and crosslinking. Product consistency and reproducibility are thereby improved. The size and configuration of each component were calculated to maintain a uniform temperature field in the reactor. The design also considered manufacturing feasibility.

Industrial self-regulating heating cables commonly have a flat, rectangular cross-section. For example, the BARTEC HTSB self-regulating heating cable (with a braided layer and outer sheath) has dimensions of 12.1 mm × 5.4 mm, and the Danfoss RX-C self-regulating heating cable (Danfoss A/S, Nordborg, Denmark) has dimensions of 11.5 mm × 5.5 mm. Accordingly, the heating-cable cross-sectional dimensions can be denoted as $\omega_{h}\approx 11.5\sim 12.1\;\mathrm{mm}$ and $t_{h}\approx 5.4\sim 5.5\;\mathrm{mm}$. The clamp must span the heating cable while providing sufficient clearance on both sides for welding or pressing. Let the allowance on each side be e (mm); the minimum clamp length should then satisfy:

(8) $$L_{c}\geq \omega_{h}+2e$$

With a centered welding-assembly allowance of e=10 mm, substitution into $\omega_{h}\approx 11.5\sim 12.1\;mm$ yields $L_{c,min}=31.5\sim 32.1\;mm$. The $L_{c}=50\sim 80\;mm$ designed clamp length for this device is substantially greater than the minimum requirement; therefore, the clamp length is fully adequate and provides a generous manufacturing margin, which is beneficial for stable bonding and vibration resistance. To achieve a uniform wall heat flux, the design adopts a coil pitch of the same order as the heating-band width. Let the coil pitch be defined as:

(9) $$p=k\omega_{h},\;k\in [1.0,1.5]$$

Substituting $\omega_{h}\approx 11.5\sim 12.1\;\mathrm{mm}$ yields p≈11.5~18.2 mm. According to the formula for the reactor circumference:

(10) C=πD

Using the number of turns N given in Equation (11) and the single-turn helical length l given in Equation (12), the total heating-wire length for N turns is $L_{h}$, as shown in Equation (13). With clamps distributed uniformly along the heating wire at a spacing s, the required number of clamps M is calculated as shown in Equation (14).

(11) $$N=\left[\frac{H}{p}\right]$$

(12) $$l=\sqrt{p^{2}{+C}^{2}}=\sqrt{p^{2}{+\left(\pi D\right)}^{2}}$$

(13) $$L_{h}=Nl=\left[\frac{H}{p}\right]\sqrt{p^{2}{+\left(\pi D\right)}^{2}}$$

(14) $$M=\left[\frac{L_{h}}{s}\right]+1$$

For this device, the heating-zone height H≈500~800 mm is specified, and substituting the resulting p≈11.5~18.2 mm into Equation (11) yields N=35~58 turns. The reactor circumference C=942.48 mm is calculated using Equation (10) and substituted into Equation (12) to obtain l, after which $L_{h}=32.9\sim 54.58\;m$ is determined from Equation (13). Finally, Equation (14) yields the upper and lower bounds for the number of clamps, $M_{min}=\left[\frac{L_{h}}{150}\right]+1$ and $M_{max}=\left[\frac{L_{h}}{250}\right]+1$. Algebraically, the minimum and maximum numbers of clamps were calculated to be 189 and 315, respectively. Because a smaller spacing increases the number of clamps—improving adhesion stability but increasing manufacturing labor—these results indicate that the device designed in this study satisfies manufacturability requirements in a TRIZ-guided design framework.

### 3.4. Comparison of Temperature Control in Gel Devices

The nucleation, polymerization, and crosslinking processes of nanogels are highly sensitive to temperature. Minor temperature fluctuations or spatial gradients can amplify heterogeneity in reaction kinetics. This effect influences particle size distribution and product consistency. A systematic analysis was conducted to evaluate the impact of different temperature control strategies on the reaction system. The comparison included a conventional heating system and a PTC self-limiting heating system. The analysis focused on temperature response, temperature uniformity ($\sigma_{T}$), and maximum temperature difference ($∆T_{max}$). These parameters were evaluated during the heating stage (25–40 °C) and the isothermal stage (35 °C for 2 h). The results provide a quantitative basis for optimizing reactor design and improving nanogel synthesis quality. The findings also show that the TRIZ-derived strategy based on material-level self-feedback heat input and structured heat-transfer homogenization significantly improves the temperature field in thermosensitive nanogel systems.

**Figure 9:**
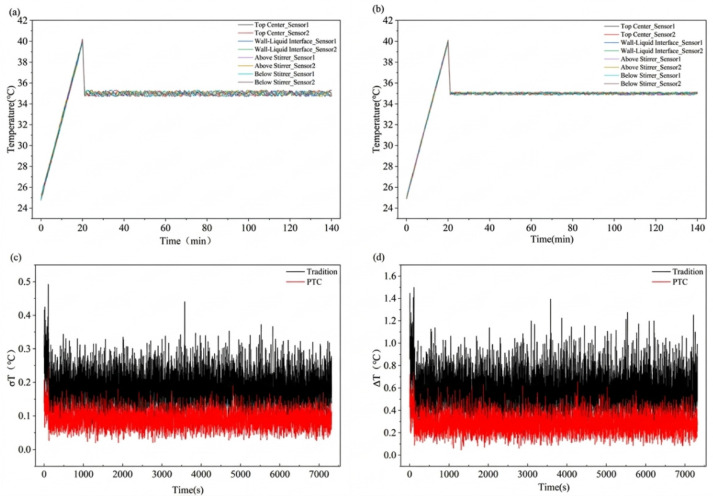
Temperature–time curves of the conventional temperature control system and the PTC heating system under a two-step temperature program: heating from 25 °C to 40 °C, followed by holding at 35 °C for 2 h: (**a**) temperature trajectories of sensors in the traditional system; (**b**) temperature trajectories of sensors in the PTC system; (**c**) $\sigma_{T}$; (**d**) $∆T_{max}$.

As shown in Figure 9a, the conventional system exhibits a pronounced thermal inertia effect during the heating stage from 25 °C to 40 °C. The temperature increases relatively smoothly and reaches the upper limit of 40 °C at approximately 60 s. The system then enters the isothermal reaction stage at 35 °C. Due to local differences in thermal inertia among the measurement points, instantaneous temperature fluctuations of ±0.3–0.5 °C occur during heating. During the isothermal stage at 35 °C for 2 h, the conventional system still shows a temperature deviation of ±0.2–0.3 °C, indicating local heterogeneity in heat transfer. By contrast, the PTC self-regulating heating system responds more rapidly, as shown in Figure 9b. During the heating stage, the PTC system reaches the upper temperature limit in approximately 50 s. It then rapidly transitions to the constant-temperature stage at 35 °C, with only small fluctuations around the plateau. Temperature fluctuations during the isothermal stage are markedly reduced. The temperature control curve remains stable and enables rapid and uniform temperature transitions. Experimental results show that the PTC system suppresses local overshoot and delayed heating through adaptive power adjustment. This feature helps maintain temperature uniformity in the polymer reaction system.

As shown in Figure 9c, the RMS profile was calculated from the measured thermocouple response curves using Equation (1). This parameter reflects the temperature uniformity inside the reactor. During the heating stage, the average value of $\sigma_{T}$ in the conventional system was approximately 0.8–1.5 °C. During the isothermal stage, the average value was approximately 0.7–1.0 °C. A peak appeared in the middle of the heating stage. This peak indicates spatial temperature differences caused by local thermal inertia. The PTC system showed clear improvement. During the heating stage, the average value of $\sigma_{T}$ was approximately 0.3–0.5 °C. During the isothermal stage, the average value was approximately 0.2–0.3 °C. The fluctuations remained stable throughout the entire process. The reduction in A indicates that the PTC system improves temperature uniformity inside the reactor. This improvement results in more consistent reaction rates at different measurement points. The controllability and uniformity of nanogel synthesis are consequently enhanced.

As shown in Figure 9d, the maximum temperature difference $∆T_{max}$, calculated using Equation (2), quantifies the temperature range among measurement points inside the reactor at a given moment. In the conventional system, the peak value of $∆T_{max}$ during the heating stage is approximately 2.5–4.5 °C. Fluctuations of about 2–3 °C remain during the isothermal stage. These variations indicate local temperature overshoot or delayed heat transfer. Such conditions may cause earlier nucleation or higher crosslinking density in certain regions. In the PTC system, the peak value of $∆T_{max}$ during the heating stage is approximately 1–2 °C. During the isothermal stage, the average value is about 0.5–1 °C. These results show a clear reduction in spatial temperature differences. The PTC system effectively suppresses local hot spots and cold spots. Temperature uniformity in the reaction environment is improved at the source. This improvement helps narrow the particle size distribution of nanogels.

The calculated $∆T_{max}$ and $\sigma_{T}$ values were further used to determine ηTS according to Equations (3)–(7), with $T_{set}$ set at 35 °C. The results are summarized in Table 6. The statistical windows for $∆T_{max}$, $\sigma_{T}$, and ηTS were uniformly defined over the 35 °C isothermal stage. Here, $t_{0}$ denotes the time at which the average temperature of the eight measurement points in the reactor first reaches and stabilizes within the 35 ± 0.5 °C plateau. The end time was defined as $t_{f}$ = $t_{0}$ + 7200 s. ηTS was then used to characterize temperature fluctuations, spatial non-uniformity, and overshoot behavior during the 35 °C isothermal reaction stage.

Table 6 shows that the PTC system outperforms the conventional system in overall temperature control performance. Specifically, the average $\sigma_{T}$ decreased from 3.9 ± 0.2 in the conventional system to 1.8 ± 0.2 in the PTC system, corresponding to a reduction of approximately 53.8%. This result indicates a significant decrease in temperature dispersion around the mean temperature at different measurement points in the reactor. The uniformity of the temperature field was also clearly improved. The average $∆T_{max}$ decreased from 6.5 ± 0.4 in the conventional system to 3.2 ± 0.4 in the PTC system, corresponding to a reduction of approximately 50.8%. This result indicates that the PTC system more effectively suppresses local hot and cold spots and reduces the spatial temperature gradient within the reactor. In addition, the temperature control performance index ηTS decreased from 0.1114 ± 0.0057 to 0.0514 ± 0.0057, corresponding to a reduction of approximately 53.9%. Because ηTS is a comprehensive indicator derived by normalizing $\sigma_{T}$ to the set temperature and averaging it over the key reaction time window, its decrease indicates that the PTC system reduces instantaneous temperature fluctuations and maintains better temperature stability throughout the reaction stage. Overall, the PTC system shows clear advantages in temperature dispersion, maximum temperature difference, and comprehensive temperature control performance. These results indicate that the PTC reaction device designed on the basis of TRIZ theory is rational and has strong application potential for improving the uniformity and stability of the reactor temperature field.

### 3.5. Reaction Mechanism and Experimental Verification

**Figure 10:**
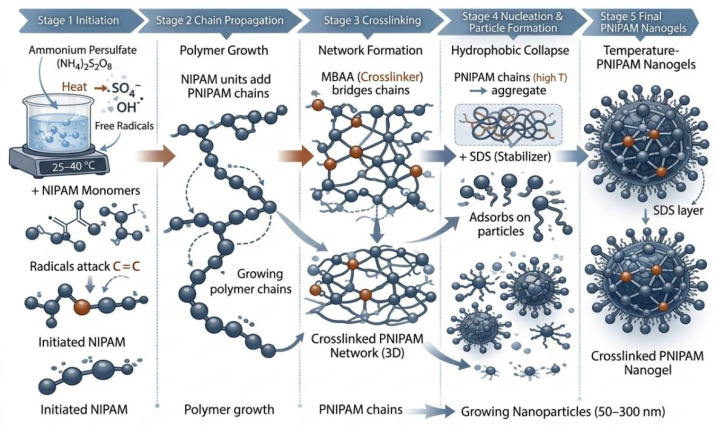
Mechanism of nanogel formation.

Based on the reaction mechanism schematic (Figure 10), thermosensitive nanogel formation proceeds sequentially through initiation, chain growth, network formation, nucleation/particle formation, and stabilization/termination, with the temperature field exerting a decisive influence during the nucleation window and the curing stage [27,28]. Spatial temperature gradients and transient overshoots can amplify variations in the free-radical polymerization rate, leading to locally premature or delayed nucleation and nonuniform crosslinking density, which ultimately broadens the particle size distribution (increased PDI) and reduces batch-to-batch consistency [29,30]. To address the contradiction between high temperature sensitivity and the difficulty of achieving a uniform temperature field, the TRIZ-designed device shifts temperature regulation from an external information closed loop to a physical-layer self-feedback mechanism: a PTC self-limiting heating element enables heating power to decrease automatically as the system approaches the target temperature range, thereby suppressing hotspot formation and overtemperature at the source. Consequently, attenuating temperature fluctuations during initiation and chain growth reduces rate oscillations, and suppressing localized overtemperature during crosslinking and particle formation inhibits premature gelation and aggregation, thereby narrowing the particle size distribution.

In this study, particle size and particle size distribution were characterized using a Malvern Zetasizer Nano ZS dynamic light scattering instrument (Malvern Panalytical Ltd., Malvern, UK). Measurements were conducted in backscattering mode with a scattering angle of 173°. The measurement temperature was maintained at 25 °C. Deionized water was used as the dispersion medium. The built-in water model of the instrument was applied for data analysis. Each sample was measured at least three consecutive times. The sample was equilibrated for 120 s before each measurement. The Z-average and PDI were determined using the cumulants method. The intensity distribution curve was used to evaluate the presence of multiple peaks or slight aggregation. FTIR characterization was performed using a Thermo Scientific Nicolet iS5 spectrometer. The spectral range was 4000–500 cm^−1^. The spectral resolution was 4 cm^−1^. Each spectrum was recorded with 32 scans. The C=C absorption band at 1620–1640 cm^−1^ was used as the diagnostic peak. The amide I band (C=O) at approximately 1650 cm^−1^ or the amide II band (N–H bending and C–N stretching) at approximately 1540 cm^−1^ was used as the internal reference. Peak areas were compared after uniform baseline correction and consistent integration ranges. The reaction conversion was estimated from the relative peak areas.

**Figure 11:**
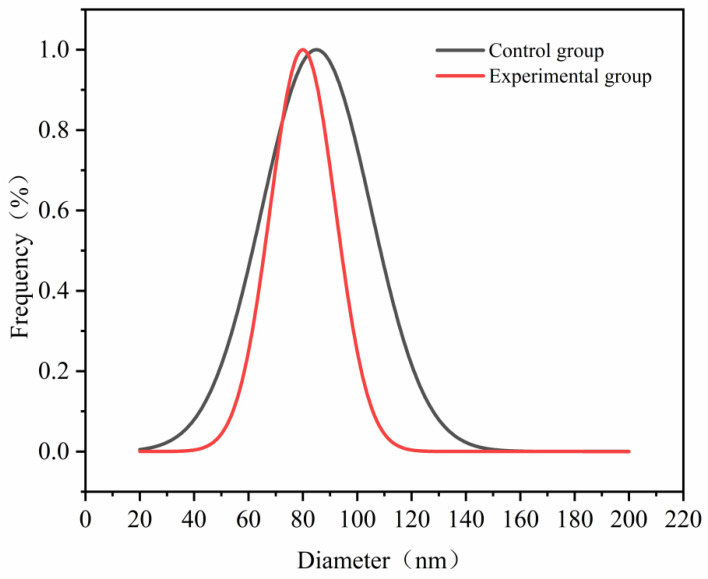
DLS particle size distribution curves of nanogels synthesized with conventional and PTC self-limiting heating devices.

As shown in the DLS particle size distribution curve (Figure 11), the main peak of the control group appears at approximately 85 nm. The distribution is relatively broad. The main peak of the experimental group shifts to approximately 80 nm. The peak becomes significantly narrower. These results indicate that the improved device slightly reduces the average particle size and produces a more concentrated distribution. This trend is consistent with the results in Table 7. The mean particle size of the experimental group decreases from 85 nm to 80 nm. The PDI decreases from 0.32 to 0.18. These results indicate that the improved temperature control strategy reduces the average particle size and improves particle uniformity. The broader intensity distribution of the control group suggests greater particle size dispersion or weak aggregation. In contrast, the experimental group shows a sharper peak and a smaller full width at half maximum. This feature reflects a more synchronized nucleation and growth process during particle formation. The dispersion stability of the system is therefore improved. Improved temperature field uniformity weakens kinetic differences among local regions during polymerization. This effect reduces variability in particle growth pathways. The observation is consistent with the TRIZ principle that improved temperature field uniformity enhances product consistency.

**Figure 12:**
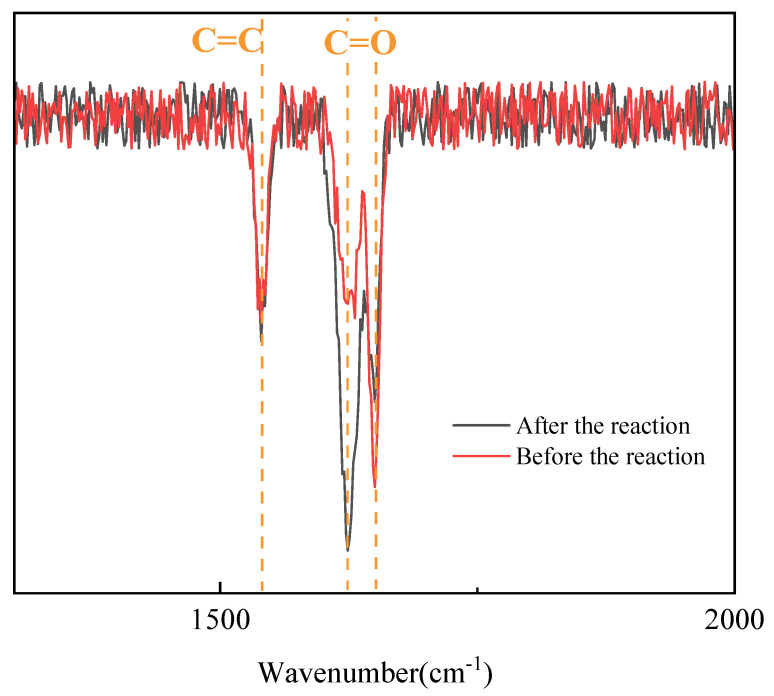
FTIR spectra of nanogels before and after polymerization using the PTC self-limiting heating device.

As shown in the FTIR spectrum (Figure 12), the C=C absorption peak at 1620–1640 cm^−1^ is prominent in the sample before the reaction. The intensity of this peak decreases significantly after the reaction. The amide I band at approximately 1650 cm^−1^ and the amide II band at approximately 1540 cm^−1^ remain clearly identifiable. These bands were used as normalization reference peaks. The results indicate that monomer double bonds are progressively consumed during polymerization. The system undergoes extensive free-radical polymerization and crosslinking. The results in Table 7 show that the conversion of the experimental group increased from 90% to 95%. This improvement indicates that the more uniform and stable thermal environment provided by the improved device promotes effective reactant conversion in the key temperature range.

Based on the combined DLS and FTIR characterization results, the improved device enhances product performance in several aspects. The results indicate more complete structural transformation and more uniform particle distribution. The DLS curve shows a narrowed particle size distribution. This feature reflects improved macroscopic dispersibility and product consistency. The FTIR spectrum indicates more complete consumption of monomer double bonds. This observation reflects an increased extent of the microscopic chemical reaction. These two observations corroborate each other. They indicate that optimizing the heat input method and improving temperature field uniformity enhance the kinetic stability of the polymerization reaction. The quality of the final product is also improved. These characterization results provide strong experimental support for the conclusion that the self-feedback temperature-limiting and structurally isothermal strategy improves the consistency and conversion efficiency of nanogels.

## 4. Conclusions

This study addresses the persistent challenge of achieving uniform temperature control in thermosensitive nanogel synthesis. By applying a TRIZ-guided, material-level self-feedback heating strategy combined with structurally homogenized heat transfer, the reactor achieves precise thermal regulation without increasing system complexity. This approach uniquely integrates physical-layer adaptation with structural optimization, providing a novel solution that surpasses conventional multi-point feedback control strategies.

(1) The optimized reactor significantly improved temperature uniformity, reducing the maximum temperature difference from 6.5 °C to 3.2 °C and decreasing the standard deviation from 3.9 °C to 1.8 °C, demonstrating enhanced spatial thermal stability. (2) The improved thermal control led to higher reaction efficiency and product quality, increasing monomer conversion from 90% to 95% and narrowing the particle size distribution, with PDI decreasing from 0.32 to 0.18. (3) Energy consumption per unit mass of reactant decreased from 3.6 kJ·g^−1^ to 2.5 kJ·g^−1^, indicating that the TRIZ-based self-feedback design improves process efficiency and provides a transferable strategy for temperature-sensitive polymerization and nanomaterial synthesis.

## 5. Patents

A Chinese utility model patent application related to the device design described in this paper has been filed and accepted by the China National Intellectual Property Administration (CNIPA; Acceptance Notice date: 10 October 2025). The application is currently under post-acceptance examination/authorization and has not yet been granted. Title: An Isothermal Reaction Device for Nanogel. Application No.: 202522139067.X (Acceptance Notice No.: 202522139067X). Filing date: 10 October 2025. Applicant: Liaoning University of Science and Technology. Inventors: Mingzhe Wang, Zhuohan Li, Xihao Wang, Dewei Hei, Yingxue Teng.

## Figures and Tables

**Table 1:** Key reagents and experimental conditions.

Category	Chemicals	Purity (%)	Manufacturer	Concentration (Per 100 mL)
monomer	N-isopropylacrylamide (NIPAM)	≥99	Aladdin Industrial Corporation (Shanghai, China)	10 mg·mL^−1^
crosslinker	N,N′-methylenebisacrylamide (MBAA)	≥99	Macklin Biochemical Co., Ltd. (Shanghai, China)	5 wt%
radical initiator	ammonium persulfate (APS)	≥98	Sinopharm Chemical Reagent Co., Ltd. (Shanghai, China)	0.5 mg·mL^−1^ (Relative to the monomer mass)
stabilizer	sodium dodecyl sulfate (SDS)	≥99	Xilong Scientific Co., Ltd. (Guangzhou, China)	0.2 mg·mL^−1^
solvent	deionized water	18.2 MΩ·cm	laboratory ultrapure water system	make up the volume to (100 mL)

**Table 2:** Summary of system function interactions.

Function Type	Actor (Component)	Action	Object	Engineering Implication
Beneficial	Agitation system (motor–stirring shaft–impeller)	Agitation/convection	Reaction medium	Enhances mixing and convective heat transfer, thereby reducing macroscopic concentration and temperature gradients.
Beneficial	External heating unit (electric heater)	Heat supply	Vessel/reaction medium	Brings the system to, and maintains it within, the target reaction temperature zone.
Beneficial	Feed line/sealed inlet	Delivery/sealing	Feedstock(s)	Enables multiphase feeding while ensuring sealing integrity and operational safety.
Harmful	Temperature sensor (measurement point)	Measurement/characterization	Temperature field	Limited representativeness of the measurement point may lead to temperature-control bias.
Harmful	Temperature-control bias	Triggers	System	Induces localized overheating/temperature gradients, affecting polymerization and causing quality fluctuations.
Side effect/limitation	Closed-loop chain (measurement–control–actuation)	Adds links	System	Increases complexity due to sensor placement, calibration, and response lag.

**Table 3:** Analysis of engineering contradictions and physical contradictions.

Category	Improving Parameter (Need Higher)	Worsening Parameter (Need Lower)	Potential Risks
Engineering contradiction	17: Temperature; 13: Stability of the object; 28: Measurement accuracy	36: Device complexity; 34: Maintainability (ease of repair); 27: Reliability	Increased dependence on measurement points; higher workload for sensor placement and calibration.
	9: Speed; 25: Time loss	31: Harmful factors acting on the object; 17: Temperature	Fluctuations in thermosensitive polymerization, leading to reduced product consistency.
	28: Measurement accuracy; 24: Information loss	36: Device complexity; 26: Amount of substance; 34: Maintainability (ease of repair)	More complex equipment, more potential failure points, and increased downtime for maintenance.
	13: Stability of the object; 27: Reliability	33: Ease of operation; 34: Maintainability (ease of repair); 36: Device complexity	Production takt/throughput may be affected; operating and maintenance costs increase.
Physical contradiction	21: Power	31: Harmful factors/overtemperature	Hotspots/thermal gradients and amplified fluctuations in thermosensitive reactions.
	28: Measurement accuracy	36: Device complexity; 26: Amount of substance	Measurements may be unrepresentative of the bulk, leading to temperature-control bias.
	17: Temperature; 13: Stability	22: Energy loss	Reduced energy efficiency and increased operating cost.

**Table 4:** System component resource table.

TRIZ Resource	Subsystem (In-Device)	System (Reactor)	Supersystem
Material	agitator; vessel/walls; feed–seal parts; valves/tubing; reaction medium; jacket/interlayer fluid; insulation/seals	reactor body/assemblies	feed supply; utilities (cooling water/steam); consumables/spares
Field	mixing/shear flow; local heat-transfer; electric-to-heat conversion	global thermal + flow fields; reaction heat	power supply; ambient heat loss; ventilation/external temperature; external heat/cold sources
Space	in-vessel zones; impeller mixing volume; wall/lid mounting areas; jacket/insulation placement; sensor/actuator locations	reactor layout interfaces	surrounding installation space; cabinet/control location; heat-dissipation paths; utility ports
Time	start-up heating; steady reaction; exotherm peak window; cooling/termination; batch changeover; idle/maintenance	batch-cycle timing	production scheduling; utility-availability windows; diurnal/seasonal temperature windows
Information	temperature signals; stirring state; valve/feed status; power/current signals	setpoints/profiles; energy data; batch QC data	SOP/formulation & specs; SCADA/logs; environmental data; historical batches/heuristics

**Table 5:** Mapping of TRIZ Inventive Principle Application.

Mapped TRIZ Inventive Principle	Application (TRIZ-Based Rationale)	Structural Design Implementation (Reactor)
1: Segmentation	Divide heating into independent thermal units for localized regulation.	Partition the reactor wall into multiple heating zones, each with an electric heater and a corresponding temperature sensor for zoned heating.
4: Asymmetry	Redistribute heat input spatially to compensate for nonuniform heat loss/heating demand.	Arrange adjustable heaters nonuniformly on the wall/jacket and tune zone-wise power based on local temperature feedback to balance the temperature field.
10: Preliminary action	Precondition heat transfer to suppress transients and reduce reliance on complex closed-loop control.	Introduce a high-thermal-conductivity jacket/thermal buffer to stabilize heat flow and temperature distribution during heating.
35: Parameter changes (physical-property transformation)	Use temperature-dependent properties to achieve adaptive (self-regulating) heat input.	Integrate PTC heaters into/on the reactor wall so power decreases automatically near the target temperature range, mitigating overshoot/hotspots and reducing dependence on external sensing.
39: Intermediate medium	Add a buffer medium to decouple external disturbances and smooth thermal fluctuations.	Add an interlayer filled with inert gas or heat-transfer oil to provide insulation/thermal buffering, improving uniformity and reducing sensitivity to ambient fluctuations.

**Table 6:** Comparison of temperature control performance.

Indicator	Conventional System	PTC System
$\sigma_{T}$ (°C)	3.9 ± 0.2	1.8 ± 0.2
$∆T_{max}$ (°C)	6.5 ± 0.4	3.2 ± 0.4
ηTS	0.1114 ± 0.0057	0.0514 ± 0.0057

**Table 7:** Comparison of control experiment data.

Measurement	Instrumentation	Experimental Group	Control Group
Maximum temperature difference ($∆T_{max}$)	K-type thermocouple + data acquisition module (NI USB-9211A, National Instruments, National Instruments Corporation, Austin, TX, USA, ±0.1 °C)	3.2 ± 0.4 °C	6.5 ± 0.4 °C
Temperature standard deviation ($\sigma_{T}$)		1.8 ± 0.2 °C	3.9 ± 0.2 °C
Reaction conversion (%)	FT-IR spectrometer (Thermo Scientific Nicolet iS5)	95 ± 1.2%	90 ± 1.2%
Z-average particle size (Dz-avg)	Dynamic light scattering (DLS; Malvern Zetasizer Nano ZS, 0.3 nm~10 μm, ±2%)	80 ± 4.0 nm	85 ± 4.0 nm
Polydispersity index (PDI)		0.18 ± 0.02	0.32 ± 0.02
Energy consumption per unit mass of reactant (kJ·g^−1^)	Power analyzer (Chauvin Arnoux PEL 103, Chauvin Arnoux, Paris, France, ±0.2%)	2.5 ± 0.4	3.6 ± 0.4

Note: The mass of the reactants refers to the total mass of the solution in the reaction system.

## Data Availability

The original contributions presented in this study are included in the article. Further inquiries can be directed to the corresponding author.
